# Surgery of the Spine

**Published:** 1896-01-18

**Authors:** 


### SURGERY OF THE SPINE.

(*Continued from page* 235.)

**Chronic Spinal Abscess.**—In a "comparative study," Albert Carless[9] reviews the present position held by surgeons as to the appropriate treatment in cases of chronic spinal abscess. Asepsis is the first essential in all cases, and it is never to be forgotten that "if a chronic abscess be opened antiseptically and kept free from contamination the resulting discharge is henceforth merely serous, and there is no rise of temperature even if a sinus remains for years." The various methods available are: (1) Aspiration, which is seldom successful in bringing about a cure, save in cases of residual abscess, in which inflammation and suppuration have supervened. The causes of failure are imperfect emptying, and therefore the leaving behind of active bacilli, and the frequent occurrence of hæmorrhage into the cavity as a result of rapidly withdrawing the pus. This blood forms a good nidus for the retained organisms, and the mischief goes on. (2) Free aseptic incision and drainage. This method often fails because, although the pus is removed, the lining membrane of the abscess is not. The difficulty of preserving asepsis is great, and the healing process takes a very long time—sometimes years. (3) Tapping with simple lavage is little better, for very much the same reasons as last. (4) Incision with removal of the pyogenic membrane, according to Watson-Cheyne,[10] succeeds in 70 per cent. of cases. He makes a small incision, and scrapes the wall with a flushing spoon, thoroughly removing every trace of the membrane. The abscess cavity is then obliterated by deep stitches; and, by appropriate pads and bandages, pressure is so arranged as to maintain the walls in apposition till healing takes place. Caird, of Edinburgh,[11] records six cases of psoas abscess treated in this method. One patient died (whether of acute septicæmia or of iodoform poisoning seems doubtful). The cardinal points which make for success seem to be (*a*) perfect asepsis; (*b*) thorough removal of the lining membrane with the finger or sharp spoon, making as many incisions as necessary for this object; (*c*) free flushing with sterile lotion to get rid of all *débris*, and to check hæmorrhage, which is sometimes very free. (*d*) Careful antiseptic dressing. (*e*) Rest in bed with a long splint. The value of iodoform seems to this writer to be doubtful. (*f*) Injection with antiseptics after emptying. This method has the support of many continental and American surgeons, notably Wieland of Basel.[12] After emptying the abscess by aspiration or tapping a 10 per cent. emulsion of iodoform (previously sterilised by washing in 1 to 20 carbolic) is injected and left in the cavity. Bandages are then applied to obliterate the sac. Senn values this method very highly. It has the drawback that toxic effects sometimes follow from iodoform. These are high temperature, diarrhœa, vomiting and violent delirium, or sometimes coma.

**Spina Bifida.**—Tansini[13] records a successful operation for spina bifida by excision. The tumour was the size of a child's head, reached from the second lumbar to the last sacral vertebra. Some nerve fibres were restored to the canal, and although healing did not take place by first intention the child was discharged well in forty days. Howitt[14] reports seven cases in which the neck of the sac was ligatured with four recoveries.

**Meningococle.**—In dealing with cranial meningocoele, Mayo-Robson[15] says the only real difficulty is that occasionally a portion of the nerve centres may be contained in the sac. This can generally, although not always, be diagnosed, and if doubt remain an aseptic exploratory incision will remove it without adding materially to the patient's risk. In most cases the sac has a distinct neck which may be ligatured, and if a flap is dissected up and replaced so that the superficial stitches and the ligatured neck of the sac do not correspond a good result may be anticipated. As an example, Mr. Robson cites the case of a healthy child, aged six months, with a large occipital meningocoele, which was becoming inflamed and the coverings thinned. A flap was raised, the narrow opening (one-third of an inch) into the skull defined, the neck ligatured, and the sac removed. The recovery was rapid and complete, and no untoward symptom had developed "some time afterwards."

**Tapping the Vertebral Canal.**—In The Hospital for October 5th, 1895, p. 10, Fürbringer's paper on tapping the vertebral canal in cases of tubercular meningitis was summarised. Caillé[16] has reported further cases in which the diagnosis was verified by this means. He taps between the third and fourth lumbar vertebræ with a large hypodermic syringe, the fluid being allowed to drop from the needle, without aspiration. The writer also values the procedure for its curative effects in conditions of increased intra-cranial tension.

[9] Practitioner, Sept., 1895. [10] System of Surgery, Treves Vol. I. [11] Edin. Hosp. Rep., Vol. III. [12] Deut. Zeitschrift für Chirurg., July, 1895. [13] Ref. Med., Aug. 6, 1895. [14] N.Y. Med. Rec., Aug., 1895. [15] Amer. Jour. Med. Sciences, Sept., 1895. [16] N.Y. Med. Jour., June, 1895.

